# Finding the right balance: aOX40 immunotherapy enhances effector T cell function without impairing regulatory T cells intrinsic suppressive capacity in mouse tumor models

**DOI:** 10.1186/2051-1426-3-S2-P77

**Published:** 2015-11-04

**Authors:** Amy Moran, Fanny Polesso, Minhazur Sarker, David Parker, Susan E Murray, Andrew Weinberg

**Affiliations:** 1 Earle A. Chiles Research Institute, Portland, OR, USA; 2 Robert W Franz Cancer Research Center, Portland, OR, USA; 3 Providence Cancer Center, Portland, OR, USA; 4 Department of Biology, University of Portland, Portland, OR, USA

## 

Expression of the co-stimulatory molecule OX40 is induced after TCR ligation on conventional T cells and the importance of OX40 in promoting proliferation, effector function, and survival of activated T cells are illustrated in multiple disease models. Paradoxically, CD4+Foxp3+ regulatory T cells (Tregs) constitutively express OX40 in mice and OX40 receptor ligation by agonist antibodies has been described to inhibit their function. Given the described role of anti-OX40 (aOX40) in decreasing Treg function and the importance of Tregs in maintaining tissue homeostasis, it's interesting to note that no dose limiting toxicities were observed in the phase I clinical trial with aOX40. Given these findings and the development of new tools to better interrogate Treg function, we revisited the idea that aOX40 immunotherapy decreases Treg function. We find that OX40 receptor ligation on Tregs does not impair their cell intrinsic suppressive capability but rather, enhances T cell effector function making them resistant to suppression.

Utilizing suppression assays, Foxp3 reporter mice, and OX40 KO effector cells, we ensured direct effects of aOX40 on Foxp3+Tregs in traditional Treg suppression assays. We highlight that aOX40 does not impair the intrinsic suppressive function of Tregs but promotes the effector function of T cells in an OX40 dependent fashion. Using two tumor models, we show that aOX40 treated Tregs are phenotypically and functionally indistinguishable from control treated cells. However, aOX40 enhanced the effector function of CD4 and CD8 T cells. Importantly, in the tumor the ratio of Tregs:T effectors decreased while the turnover of Tregs increased as compared to the periphery. *Ex vivo*, after aOX40 immunotherapy, tumor infiltrating CD4+Foxp3-cells made more TNFa (p=.0316) and IFNg (p=.0328) upon re-stimulation than control treated animals. Moreover, OX40 sufficient conventional CD4 T cells made more IL-2 *in vitro* when stimulated with aCD3 than control treated cells. Notably, despite the functional Tregs in the tumor, the enhanced T cell effector function mediated by aOX40 immunotherapy increased the survival of tumor bearing mice by 25-30% (p=.0135). Taken together, we propose that the use of Foxp3 reporter mice provided a more rigorous assessment of the mechanism of action of OX40 agonists on Treg and effector T cell function in multiple tumor models. In contrast to what has been previously published, we show that after aOX40 immunotherapy, tumor derived Treg function is not reduced. Rather, the effector function of conventional T cells is enhanced promoting increased survival in tumor bearing mice.

